# Fluid Therapy in Pulmonary Disease: How Careful Do We Need to Be?

**DOI:** 10.3389/fvets.2021.624833

**Published:** 2021-08-09

**Authors:** Sophie Adamantos

**Affiliations:** Paragon Referrals, Wakefield, United Kingdom

**Keywords:** crystalloid, colloid, pulmonary oedema, cat, dog

## Abstract

Intravenous fluid therapy is a vital and life-saving therapeutic in veterinary medicine. In the absence of heart or lung disease, trauma or sepsis there is limited evidence that fluid therapy will have a detrimental effect on lung function. In healthy dogs there is a reasonable level of experimental evidence that supraphysiologic rates of fluid are required before signs of fluid overload are made evident. In cats, however, this may not be the case. There are higher rates of asymptomatic myocardial disease, but even in the absence of that it seems that some cats may be susceptible to fluid overload. Where systemic inflammation already exists the careful homeostatic and protective mechanisms within the lung are deranged and increases in hydrostatic pressure are more likely to result in fluid movement into the lung tissues. Strategies including restricting the use of intravenous crystalloid fluid administration and using blood products for management of severe hemorrhage are of increasing importance in human trauma and seem to be associated with fewer pulmonary complications, and lower mortality. Managing dogs and cats with sepsis and acute respiratory distress syndrome is already challenging, but ensuring adequate vascular expansion needs to be balanced with avoiding excessive volume administration which may negatively impact pulmonary function. While fluids remain crucial to management of these conditions, there will be an ongoing requirement to balance need without providing excess. The use of point of care ultrasound may provide clinicians with a non-invasive and accessible way to do this.

## Introduction

Fluid therapy is a vital part of the management of the acutely or critically ill veterinary patient. Despite being considered a relatively benign therapy in veterinary practice, emerging evidence in human patients has identified that there is a need for careful fluid balance to avoid excessive morbidity or mortality (1). This effect seems to be most pronounced in the most severely ill people. An association between increased mortality and cumulative fluid balance in people with acute respiratory distress syndrome (ARDS) has been shown (2). In healthy animals the lung is protected against the effects of excessive fluid accumulation. However, in disease where there may be alterations in fluid fluxes, these protective strategies may fail, and excessive fluid administration can significantly affect lung function. This article will discuss normal fluid dynamics in the lungs, the influence of disease on these dynamics, and the potential impact of fluid therapy. The following writings will explore the experimental, human and veterinary clinical evidence specifically related to conditions where fluid therapy and pulmonary dysfunction are of particular concern; trauma and pulmonary contusions, sepsis, systemic inflammatory response syndrome (SIRS) and acute respiratory distress syndrome (ARDS).

## Fluid Dynamics in The Lung

Fluid dynamics within tissues are complex. In recent years the endothelial glycocalyx has been identified as an important consideration in the formation of extravascular fluid, as well as the forces described by Starling (3, 4). Starling's original model defined the movement of fluids from the semi-permeable intravascular spaces as being subject to intra- and extravascular colloid osmotic pressure (COP) and hydrostatic forces, specifically the gradients formed between these at the arterial and venous ends of the capillary. Starling's model proposed that the majority of fluid extravasation occurring at the arterial end and fluid resorption at the capillary end. In health the glycocalyx, which is a thin matrix of glycoproteins, proteoglycans and glycosaminoglycans on the endothelial (luminal) surface of vells, maintains normal vascular permeability and transvascular fluid movement. The effect of the glycocalyx is that, in contrast with the original Starling model, the COP contributes less of an impact to transvascular fluid flux. In the revised model the COP gradient is generated between the plasma (within the vessel) and the subglyceal space. The COP of the subglyceal space is very low, and the effect of this is that the most relevant COP gradient is between the endothelial surface layer and the subglyceal space rather than the lumen and the subglyceal space. As a result of this fluid extravasation is low and constant along the entire length of the capillary and in contrast with Starling's original model the resorption of fluid is insignificant (5, 6). Damage to the glycocalyx, as measured by increased shedding of glycocalyx biomarkers, occurs in several disease states including trauma and sepsis. It is thought that this damage results in a loss of integrity and increased vascular permeability and fluid flux. It is also thought that subsequent to the loss of glycocalyx the forces described by Starling and in particular hydrostatic pressure, are of greater importance (7). As hydrostatic pressure can be increased through administration of fluid therapy this can potentially promote excessive fluid movement resulting in edema. The loss of the endothelial glycocalyx also results in extravasation of large macromolecules into the interstitium altering the COP gradients and making the most important COP gradient that between the plasma and the interstitial fluid.

In healthy lung tissue there are several additional protective measures that limit the formation of extravascular lung water (EVLW) (8). Capillary hydrostatic pressure can increase without significant leakage into the alveolar tissue. The pulmonary interstitium is non-distensible and limits accumulation of EVLW. This drives any fluid that does accumulate toward the lymphatics which are able to significantly increase flow in response. In addition, the formation of colloid osmotic pressure gradients when fluid transudation does occur ensures that fluid is removed. While these mechanisms function well in health, in diseases where there are alterations in microvascular permeability such as pneumonia or ARDS they are easily overwhelmed. Indeed, once vascular leak has started it is difficult to stop, thus leading to the accumulation of excessive EVLW and pulmonary dysfunction.

## Fluid Therapy and Pulmonary Function

When there is pulmonary inflammation or damage to the glycocalyx, fluid therapy affects these delicate relationships by increasing capillary hydrostatic pressure. In these circumstances the result can be overwhelming, resulting in alveolar flooding and pulmonary edema. The impact of fluid therapy and specifically that of the volume, or dose administered, is therefore of most importance in animals where there is endothelial damage or inflammation. There is limited data that supports the formation of non-cardiogenic pulmonary edema in veterinary species as a result of appropriate doses of intravenous fluid in the absence of lung injury or damage. Experimental data in healthy dehydrated dogs examining the dose response effect of lactated Ringer's solution identified that high rates and doses were associated with clinical signs of fluid overload. However, the doses that resulted in side effects associated with fluid overload were between 270 and 360 mL/kg/h administered for an hour, which would not be used in clinical circumstances (9). Furthermore, doses of 135 mL/kg administered for 60 min were not associated with pulmonary dysfunction when given under experimental conditions in dogs (10).

In veterinary patients, fluids are used for management of several conditions. In the acute situation intravenous fluids are used most commonly for management of intravascular fluid deficits (11). Management of intravascular volume deficits (hypovolemia) typically involves the administration of aliquots (25% of blood volume) of isotonic crystalloid fluids up to a rate of 90 mL/kg/h, which is below the experimental doses reported (11). Recent experimental work in a rat hemorrhage model comparing the effects of a 1:1 blood volume lost to replacement crystalloid ratio with a 1:3 ratio identified increased histologic lung edema scores and wet:dry lung weight, but there was no effect on PaO_2_ (12). Despite this, it would seem that clinically, rates used in the acute setting for resuscitation have limited immediate effect on pulmonary function in the absence of pre-existent pulmonary inflammation or injury. In the chronic phase of fluid therapy however, if these drugs are used in inappropriate circumstances, at inappropriate rates or for too long of periods, they will contribute to volume overload and a positive fluid balance. Fluid overload, defined as accumulation of edema secondary to excessive retention of administered fluid, has been identified in critically ill dogs, and has been associated with increased mortality (13). The effect of fluid overload on pulmonary function has not been evaluated clinically in dogs and remains an area in which veterinary clinicians are dependent on interpretation of the human and experimental literature. Fluid overload resulting in pulmonary dysfunction has, however, been described in a population of cats with urethral obstruction (14). In this population, heart disease (hypertrophic cardiomyopathy and hypertrophic obstructive cardiomyopathy) was identified in most of the cats (83%) in which cardiac evaluation was performed. Administration of a fluid bolus was also identified as a risk factor for fluid overload (14). As a proportion of cats will not have easily identifiable signs of myocardial disease on clinical examination (15) cats are at particular risk of fluid overload. However, it appears that clinically, even cats without heart disease are more susceptible to the pulmonary effects of fluid therapy and so extra care needs to be taken in this species.

When considering the impact of fluid therapy on pulmonary function for an individual patient, it is important to first identify the reasons for administration of fluid and know which measures you can be used to monitor effectiveness of the fluid administered. Secondly, it is important to have methods for monitoring for the development of pulmonary dysfunction and pulmonary edema. Methods for assessment of fluid requirements are poorly evaluated in the veterinary literature. We are currently reliant on physical examination, calculated indices such as shock index and biomarkers, such as lactate to identify significant hypoperfusion (16, 17). There is increasing focus on the use of point-of-care ultrasound to provide more objective measures which can be used alongside other indicators to improve and refine our approach to this (18, 19).

Pulmonary function can be monitored subjectively through the use of physical examination and using subjective measures such as pulse oximetry (more useful in anesthetised patients) or arterial blood gas analysis. Assessment for pulmonary edema can be more challenging. Techniques that measure EVLW are not available in clinical veterinary practice and are invasive. Thoracic imaging, including radiography and ultrasound are easily available and generally non-invasive. Assessing animals for the development of pulmonary edema during clinical care has been improved by the advent of point-of-care ultrasound. Several studies evaluating the use of focussed protocols for lung ultrasound have been published in veterinary medicine (20–22). This technique has also been shown to be valuable in dogs without radiographic changes (20). Identification of pulmonary infiltrates is relatively easy with minimal training and allows bedside, non-invasive assessment of cats and dogs that are considered at risk of development of pulmonary edema (23). Good correlation has been shown between the presence of B-lines and extra-vascular lung water in critically ill human patients (24). B-lines are comet tail artifacts that are visible on ultrasound of the lung tissue. They are hyperechoic, well-defined and should move synchronously with lung sliding. Less than 3 B-lines/field of view is considered normal. The use of the described lung ultrasound protocols ensures full evaluation of the lungs and therefore understanding of whether B-lines, if present, are diffuse or focal. Diffuse B-lines would be suggestive of pulmonary edema, particularly if acute in origin. Other differentials for diffuse B-lines include pulmonary fibrosis. Focal B-lines may indicate focal consolidation due to pneumonia, hemorrhage or contusion. While the published veterinary literature currently focusses on the use of detection of B-lines in dogs and cats with pulmonary edema secondary to congestive cardiac failure, there is no reason while the same ultrasonographic approach cannot be used for the monitoring of patients at risk of non-cardiogenic pulmonary edema.

## Pulmonary Effects of Fluid Therapy in Trauma and Lung Contusions

The detrimental effects of intravenous fluid therapy on lung contusions have been known for over 60 years (25). An association between high volumes of fluid following trauma and mortality was identified nearly 30 years ago, although causation was not shown (26). Resuscitation in trauma remains controversial in human patients and is an area of active research. More recently there has been increased focus on the use of blood products for definitive volume replacement, particularly when there is massive blood loss (27).

Experimental studies in pigs in a model of blunt lung injury have confirmed a relationship between increased lung water and administration of crystalloid or artificial colloid solutions, with variable clinical effects (28–31). In these studies, use of crystalloid solutions were associated with increased lung water compared with colloid/crystalloid mixtures.

In an older animal model of lung contusion and hemorrhage the infusion rate of Ringer's lactate solution used for resuscitation was directly related to the degree of pulmonary insufficiency identified (31). Clinically relevant rates of 60–90 ml/kg/h were included in this study. However, rates of up to 30 mL/kg/h for a maximum of 3 h had limited effects on lung function (31). The utility of these experimental studies is questionable due to short follow up times, the cause of injury and controlled nature of the studies (28–31). However, they point toward a link between increased volumes of crystalloid administration and increased EVLW and potentially pulmonary dysfunction (28–31).

Large multi-center clinical studies in people have also identified an association between severity of hypoxemia and the volume of crystalloid administered, particularly if administered within the first 6 h following arrival at the hospital (32). Furthermore, in severely injured people predicted to require massive transfusion, an association between the development of ARDS and number of 500 mL isotonic crystalloid boluses was identified, which was independent of the volume of blood products administered. In this population, patients developing ARDS received a mean of 12 × 500 mL (total 6 liters) boluses compared with 8 × 500 mL (total 4 liters) boluses in the group that did not develop ARDS (33). In an average 70 kg person this represents a difference between 85 and 57 mL/kg. In severely injured people presenting with significant blood loss, administration of blood products during resuscitation is associated with lower mortality (34) and the current focus is on restriction of crystalloids following admission.

Applying this evidence to veterinary patients is challenging. Our patient population generally has less significant injury, or potentially our capability to manage severe injury is less than in human medicine. Availability of large volumes of blood products is also more limited in all but the largest hospitals. However, current evidence supports the use of restricted volumes of crystalloid at rates of <30 mL/kg/h to limit pulmonary effects, while ensuring adequate resuscitation using physical examination markers of perfusion, lactate concentrations and other markers of perfusion. When there is severe hemorrhage requiring fluids for resuscitation, the use of blood products instead of non-sanguineous fluids is justified where possible.

## Pulmonary Effects of Fluid Therapy in Sepsis, Systemic Inflammatory Response Syndrome, and Acute Respiratory Distress Syndrome

Fluid therapy in human sepsis is a very controversial topic despite decades of research. Following the publication of improved outcomes with early goal directed therapy large volumes of fluid were recommended to target specific blood pressure and central venous pressure end-points (35). However, a recent metanalysis did not identify mortality differences between lower and higher fluid volumes in the initial management of sepsis (36). Notably a study in African children with sepsis showed that a more restrictive fluid administration protocol resulted in improved survival (37). Perhaps not surprisingly, as sepsis is the main cause of ARDS in people (38), much of the clinical literature examining effects of the pulmonary function in critically ill human patients examines it through the lens of ARDS. Differentiation of ARDS from other causes of pulmonary edema can be challenging clinically (39). In people and veterinary species ARDS is caused by increased pulmonary capillary permeability secondary to inflammation and is defined as the presence of bilateral lung infiltrates in the absence of systolic heart failure or fluid overload. Acute respiratory distress syndrome is associated with increased EVLW, and the alveolar fluid that accumulates is typically protein rich. As there is increased pulmonary capillary leak in ARDS it follows that increases in capillary hydrostatic pressure will result in worsening of fluid accumulation. Pulmonary edema caused by left sided congestive cardiac failure (i.e., circulatory volume overload) is a contraindication for intravenous fluid therapy and will not be further discussed in this article.

**Figure 1:**
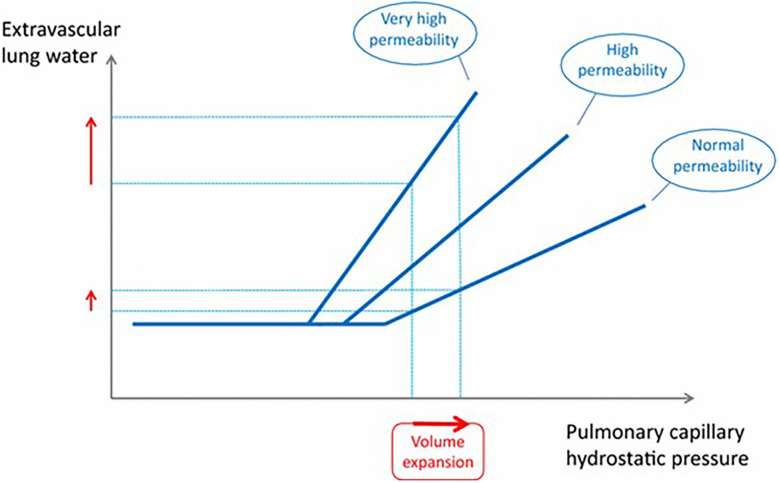
This graph shows the relationship between pulmonary capillary hydrostatic pressure and extravascular lung water. Beyond a certain point continued volume expansion results in increased extravascular lung water, an effect that is more pronounced when lung permeability is increased. From Jozwiak et al. (44).

In an experimental pig model of sepsis resuscitation with large volumes of fluid in early sepsis did not result in increased EVLW (40). Similarly in a recent retrospective study in people with sepsis who received a minimum of 30 mL/kg isotonic crystalloid fluid for resuscitation, administration of 30 mL/kg within the first 2 h, compared with lower rates of administration did not result in increased rates or duration of mechanical ventilation (40). Interestingly in this population the percentage of patients requiring mechanical ventilation was significantly lower in patients receiving the higher rates of fluid administration (0.25–0.5 mL/kg/min) (41). When specifically examining a small cohort of critically ill people that develop ARDS, a significant role for the volume of fluid administered intraoperatively has been identified, with patients receiving >20 mL/kg/h having 3.8 times higher adjusted odds of developing ARDS compared with those that received <10 mL/kg/h (42). In contrast to this, when examining risk factors for development of pulmonary edema following fluid loading in critically ill patients, relatively high baseline cardiac index and pulmonary vascular filling were found to be more important than pulmonary vascular permeability and filling pressures or volume of fluid administered (43). It was thought that these indicated a plateau in cardiac function and an inability to cope with the fluid volume administered. These effects are demonstrated in Figure 1. Even with normal permeability, when volume expansion reaches a critical point, extra-vascular lung water increases. In ARDS patients who have altered vascular permeability, these changes are seen at lower filling pressures (lesser volume expansion). In addition to volume of fluid administered, the presence of sepsis and volume of fluid administered was not found to be predictive of formation of pulmonary edema in this latter study. Although the results of these two studies may seem conflicting, one is retrospective examining patients presenting with ARDS to an ICU, and one prospective, primarily designed to examine the effect of fluid types on EVLW /pulmonary edema formation rather than the mechanisms. The studies were also measured over different time courses. In the former study the mean time to the development of ARDS was 2.6 days, whereas in the latter study pulmonary edema was inferred from increased EVLW during the course of fluid administration. It would seem therefore that in the acute setting cardiac function is more important for the formation of pulmonary edema, but that high volumes of fluid administered intraoperatively may impact on the development of ARDS.

Once ARDS is present, the management of fluid therapy becomes complex. As seen previously, many patients with ARDS have sepsis, and management of sepsis typically requires administration of intravenous fluids to maintain homeostasis. Assessing fluid requirements, responsiveness and balance is of particular challenge in these patients and an area of active research. Given that ARDS is a form of non-cardiogenic pulmonary edema associated with alterations to pulmonary vascular permeability, it makes biological sense to limit increases in hydrostatic pressure to minimize further worsening of pulmonary function. Restrictive fluid therapy strategies in ARDS are supported by several retrospective studies (45–47). A large prospective study comparing a restrictive and liberal fluid therapy approach in patients with acute lung injury (now included within the definitions of ARDS) demonstrated that the restrictive group had more ventilator free days and better lung function than the liberal group although there was no difference in outcome (48). Management of ARDS is challenging in veterinary patients, and as prolonged ventilation is both difficult and expensive how veterinarians apply the human literature in this area needs careful consideration. Avoiding deterioration of lung function is of great importance, and lessons from the human literature highlight the use of carefully balanced fluid therapy, to ensure adequate tissue perfusion while avoiding fluid overload. Clinically dogs with pneumonia may have sepsis and are at risk of ARDS (49). Pneumonia by definition represents inflammation of the lung tissue and will therefore result in alterations in fluid dynamics within the lung. It is this cohort of patients that pose the greatest challenge to veterinary clinicians, as managing their fluid requirements may impact their pulmonaryfunction.

## Discussion

Intravenous fluid therapy is a vital and life-saving therapeutic in veterinary medicine. The evidence supporting a detrimental impact of fluid therapy on lung function is limited to disease states. The effects of excessive fluid therapy in animals with known cardiac disease is well-known. In addition to this, animals that have suffered trauma, have sepsis or inflammatory lung disease should be considered at risk of deteriorating lung function as a result of overzealous fluid therapy after initial resuscitation.

The effects of fluid overload in healthy animals usually only occur in supraphysiological doses, however due to their smaller blood volume and increased incidence of undiagnosed or asymptomatic myocardial disease, extra caution is required in cats.

In sick animals the impact of the underlying condition should be considered as this may impact on the fluid therapy plan and influence monitoring.

There is clear evidence in the literature that limiting fluid rates in people with trauma is important to prevent respiratory complications. In patients with sepsis the data is more difficult to interpret. Restoration of perfusion is of critical importance, and in patients that are fluid deplete administration of intravenous fluid therapy is important, but overload must be avoided. Early use of vasopressors is increasingly promoted in people with sepsis to avoid fluid overload. The importance of this in veterinary species is unknown, and clinical experience suggests that fluid deficits are common in these patients. Assessing whether fluid deficit is present is potentially challenging, but the use of point-of-care ultrasound using for example, measurements of the caudal vena cava is allowing more careful and individualized patient care. In addition, the use of monitoring tools such as body weight, serial physical examination, urine output and specific gravity allows individualized patient care and may improve prescription of fluid therapy.

While management of these animals will remain challenging when formulating individualized and flexible fluid therapy plans, using phased approaches, e.g., ROSE approach (resuscitation, optimization, stabilization, and evacuation) will allow veterinary clincians to limit any potentially detrimentaleffects.

## Author Contributions

The author confirms being the sole contributor of this work and has approved it for publication.

## Conflict of Interest

SA is employed by Linnaeus Veterinary Limited which is a part of Mars Veterinary Health.

## Publisher's Note

All claims expressed in this article are solely those of the authors and do not necessarily represent those of their affiliated organizations, or those of the publisher, the editors and the reviewers. Any product that may be evaluated in this article, or claim that may be made by its manufacturer, is not guaranteed or endorsed by the publisher.
